# Bilateral Asynchronous Renal Cell Carcinoma with Metastatic Involvement of the Tongue

**DOI:** 10.1155/2012/729642

**Published:** 2012-09-12

**Authors:** Naseem Ghazali, Charlotte Davis, A. W. Barrett, John V. Tighe

**Affiliations:** ^1^Maxillofacial Unit, Queen Victoria Hospital NHS Foundation Trust, Holtye Road, East Grinstead RH19 3DZ, UK; ^2^Regional Maxillofacial Unit, Aintree University Hospital, Lower Lane, Liverpool L9 7AL, UK; ^3^Department of Histopathology, Queen Victoria Hospital NHS Foundation Trust, Holtye Road, East Grinstead RH19 3DZ, UK

## Abstract

Renal cell carcinoma (RCC) has a propensity for distant organ metastasis and late recurrence, involving not only the ipsilateral but also contralateral kidney. Lingual metastasis by RCC is rare. We present an unusual case of bilateral asynchronous RCC. Involvement of the right kidney was discovered only after a metastatic tongue lesion was diagnosed. The original RCC had been treated by left nephrectomy 14 years previously. Due to end-stage primary pulmonary malignancy, and poor function of the remaining kidney, immunotherapy was unsuitable. Palliative local resection of the lingual metastasis alleviated functional difficulties and was preventative against airway obstruction, but the patient died five months later.

## 1. Introduction

The oral mucosa is rarely involved by metastatic cancer. In the mouth, the tongue is the second most commonly affected subsite, with an estimated postmortem incidence of 0.2% [1]. Lingual metastasis by renal cell carcinoma (RCC) is exceedingly rare, but isolated case reports are documented sporadically [2–4]. Most cases of lingual metastatic RCC occur within 5 years of primary diagnosis. 

We present an unusual case of bilateral asynchronous RCC with lingual involvement, with an interval of 14 years between diagnosis of the original and contralateral RCC. 

## 2. Case Report

**Figure 1:**
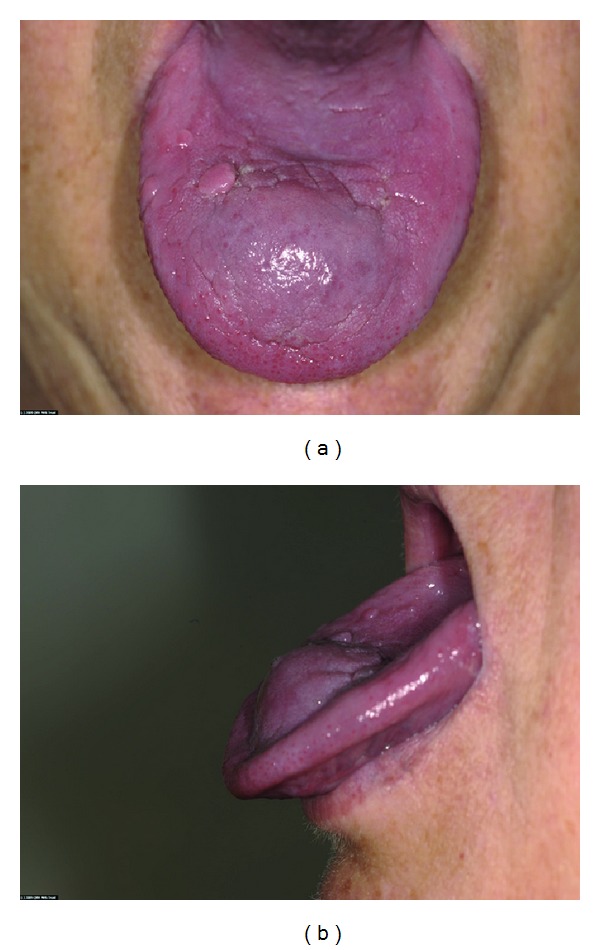
Tumour at first presentation. Painless, firm lump with no associated inflammation.

A 64-year-old woman presented with a painless lump on the anterior tongue, growing rapidly in four weeks, causing progressive functional difficulties. She also had end-stage pulmonary small cell carcinoma and was wheel chair bound. A firm, well-defined nontender lump (5 cm diameter) was found in the midline anterior tongue, just deep to mucosa (Figure 1). 

**Figure 2:**
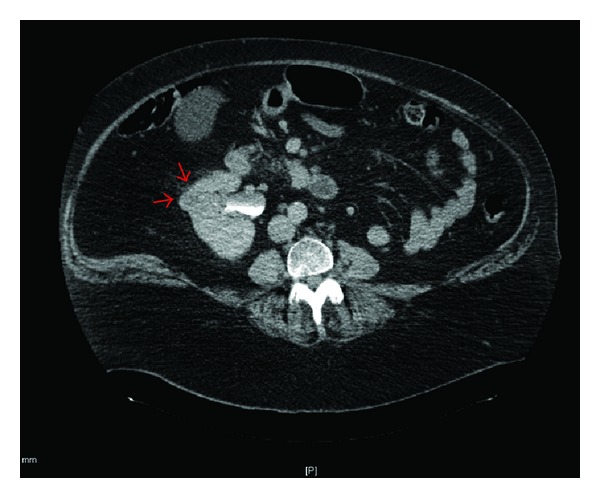
Nonenhancing hypodense mass in the anterolateral cortex of right kidney and absence of left kidney from previous nephrectomy.

Histopathological examination of an incisional biopsy showed the tumour to be composed of polygonal cells with clear cytoplasm lying in a richly vascular stroma. Immunohistochemistry was positive for a pan-cytokeratin antibody (AE1/AE3), epithelial membrane antigen, vimentin, and CD10, and negative for RCC antigen, cytokeratins 7, 19 and 20, and TTF-1. This was suggestive of a clear cell variant of RCC. When informed of the histopathological findings, the patient recalled having had left RCC (clear cell, G3, pT3a) treated elsewhere by nephrectomy in 1995, having forgotten initially due to the 14-year interval. There was no family history of hereditary RCC. Subsequent computerised tomography (CT) staging confirmed not only the absent left kidney, but also an unexpected presence of a nonenhancing, hypodense 1.6 cm mass on the anterolateral cortex of the right kidney consistent with RCC (Figure 2). Retrospective review of the imaging and histopathology of the original left RCC was not performed because the scans and slides were untraceable. 

**Figure 3:**
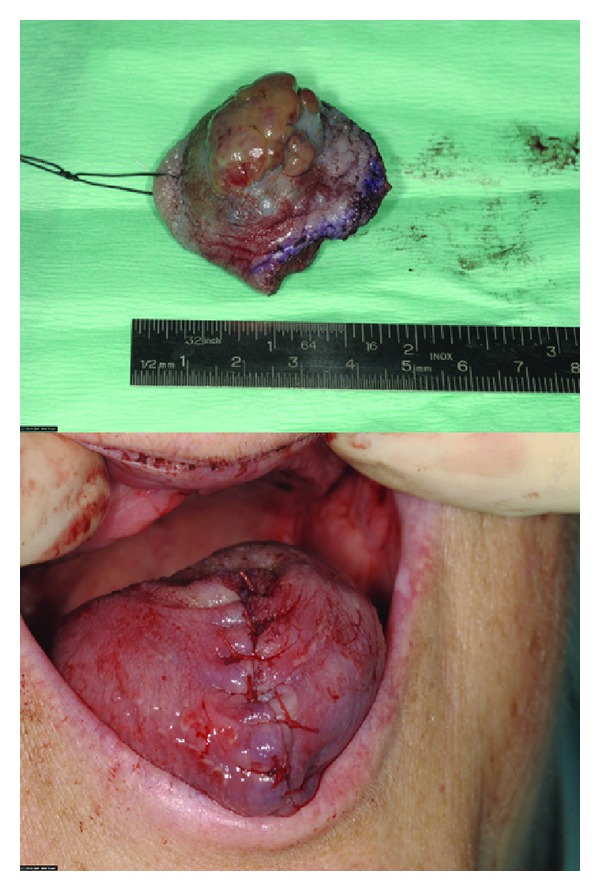
Local surgical procedure.

**Figure 4:**
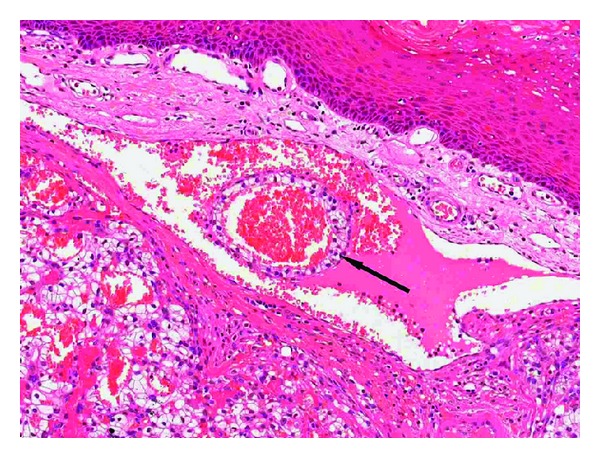
The tumour comprises polygonal cells with clear cytoplasm arranged as alveoli, separated by thin fibrous septa, around a richly vascular stroma. The presence of a “ring” of neoplastic clear cells (arrow)within a dilated capillary indicates intravascular invasion. Haematoxylin and eosin, original magnification ×100.

She was unsuitable for immunotherapy, due to relatively poor function in the remaining kidney and the end-stage pulmonary small cell carcinoma. Local resection of the metastatic tongue lesion was performed, providing symptomatic relief and to preempt potential airway obstruction by a rapidly growing lesion. The standard surgical approach was modified only by measures taken in anticipation of the haemorrhagic risk associated with RCC. Wedge excision using cutting diathermy and primary closure was performed under general anaesthesia without complications (Figure 3). Histological examination of the surgical resection specimen confirmed the initial incisional biopsy findings, with evidence of intravascular invasion (Figure 4). Following an uneventful recovery, she died five months after diagnosis.

## 3. Discussion

RCC shows propensity for multiple distant site involvement via haematogenous spread. Overt metastasis is observed in 20–30% of patients at first presentation [5]. RCC is also known for late recurrence; lesions can appear 10 years or more after initial surgical treatment [6]. Intrarenal sites can be affected by late RCC recurrence, but involvement of contralateral or bilateral renal involvement is very unusual, especially after a long interval [6]. Bilateral renal involvement, without a hereditary component, is infrequent (3–5% of cases), but tends to be metachronous, occurring within 10 years of primary diagnosis and treatment. Presence of undetectable micrometastasis at the time of original diagnosis may account for this [6]. 

Contralateral kidney involvement by the same tumour 10 years after original diagnosis may be considered a late recurrence, but could also represent *de novo* pathology. It is difficult to distinguish between the two possibilities on clinicopathological features alone. However, presence of multiple foci of RCC within the second kidney is suggestive of latent metastases rather than a new primary. The prognosis of latent, metachronous RCC is not significantly different to unilateral renal involvement, suggesting that despite a tendency for recurrence, these tumours grow slowly [6]. However, when lingual metastasis is present in the background of a latent metachronous lesion, prognosis is generally very poor because there is usually widely disseminated RCC. There are only two other reports of lingual metastases occurring more than 10 years after the diagnosis of the primary RCC [7, 8]. The case presented here is the first of a solitary lingual RCC metastasis occurring concurrently with RCC in the contralateral kidney, with a 14-year interval from the diagnosis of the primary lesion. Comparative assessment of the lingual metastatic RCC lesion with the original left kidney RCC lesion would have been valuable. However, the left kidney pathological material was unobtainable for this purpose as the surgical resection was performed in a different hospital and this may be considered one limitation of this paper.

The most common sites of metastatic RCC involvement are pulmonary (75%), regional nodal basin (65%), bone (42%), and liver (41%) [3, 4]. The head and neck and involved in 15% of cases, paranasal sinus being the most common subsite [2, 4]. Lingual involvement is often reported in the presence of other metastatic sites, especially bones [4] and lung [2]. Rarely reported as a single metastatic focus, the presence of metastatic lingual RCC highlights the possibility of extensive metastatic involvement. This was not observed in our case. 

The tongue base is the most common lingual site of involvement, due to its relative immobility and rich blood supply [3]. Metastatic involvement of the anterior tongue, as seen in our case, whilst previously reported by others, occurs much less commonly [2, 3]. The predilection for the tongue may be related to its complex vascular anatomy [9]. Production of tumour-induced cytokines in RCC, including angiogenic factors, mediators of extracellular matrix remodelling, and those that promote escape from immune-surveillance, may underlie its propensity for haematogenous spread [5]. These cytokines may also explain the bleeding tendency related to metastatic RCC foci. 

Lingual metastasis often presents as a firm, painless swelling without accompanying inflammation [10], as in our case. Other reported symptoms of metastatic lingual RCC, such as dysphagia and dysarthria, may reflect the mass effect. Others are due to the tumour's rich vascular network including a pulsating mass, violaceous appearance, and haemorrhagic surface [3]. A whole-body staging scan is recommended in the presence of lingual metastasis to evaluate multiple organ involvement. In our case, imaging revealed presence of occult RCC in the contralateral kidney. CT is the modality of choice for detection, diagnosis, and image surveillance of renal cortex tumours [4]. 

The differential diagnosis of clear cell tumours in the head and neck includes neoplasms of salivary and odontogenic origin as well as metastases. Amongst the former, (hyalinising) clear cell carcinoma (NOS), clear cell oncocytic carcinoma, and mucoepidermoid carcinoma must be excluded. Clear cell odontogenic carcinomas usually show ameloblast-like palisading, if only focally. The morphology of the present tumour, namely, aggregates of clear cells with a delicate vascular network and arranged in an alveolar pattern around pools of extravasated erythrocytes (Figure 4), is typical of RCC [10, 11]. Furthermore, expression of vimentin and lack of cytokeratins 7 and 19 virtually rule out a salivary or odontogenic origin. Whilst the lack of expression of RCC antigen may appear anomalous, up to a third of metastatic RCC are negative for this marker [12].

Surgical resection, ranging from partial to complete nephrectomy, remains the standard of care in localised intrarenal RCC. Presence of metastatic RCC after surgery suggests a poor prognosis. Immunotherapy, that is, cytokine treatment with administration of interleukin-2 and interferon-alpha, is the subsequent treatment of choice and can produce prolonged benefit [5]. However, the response to such treatment shows individual variation including the occurrence of severe cytokine therapy-induced toxicity. Clinical trials report that cytokine therapy is only beneficial in those with favourable prognostic indicators [5]. Understanding molecular events mediating the biological behaviour of RCC, including the propensity to metastasise, has introduced the real possibility of targeted therapy as a viable treatment option. Results from recent phase III clinical trials using tyrosine kinase inhibitors have shown promising results, with improvement in progression-free survival in those with metastatic RCC.
